# Generation of Neutralizing Monoclonal Antibodies against a Conformational Epitope of Human Adenovirus Type 7 (HAdv-7) Incorporated in Capsid Encoded in a HAdv-3-Based Vector

**DOI:** 10.1371/journal.pone.0103058

**Published:** 2014-07-23

**Authors:** Minglong Liu, Xingui Tian, Xiao Li, Zhichao Zhou, Chenyang Li, Rong Zhou

**Affiliations:** State Key Laboratory of Respiratory Disease, Guangzhou Institute of Respiratory Disease, The Affiliated First Hospital of Guangzhou Medical University, Guangzhou, China; Public Health Agency of Canada, Canada

## Abstract

The generation of monoclonal antibodies (MAbs) by epitope-based immunization is difficult because the immunogenicity of simple peptides is poor and T cells must be potently stimulated and immunological memory elicited. A strategy in which antigen is incorporated into the adenoviral capsid protein has been used previously to develop antibody responses against several vaccine targets and may offer a solution to this problem. In this study, we used a similar strategy to develop HAdv-7-neutralizing MAbs using rAdMHE3 virions into which hexon hypervariable region 5 (HVR5) of adenovirus type 7 (HAdv-7) was incorporated. The epitope mutant rAdMHE3 was generated by replacing HVR5 of Ad3EGFP, a recombinant HAdv-3-based vector expressing enhanced green fluorescence protein, with HVR5 of HAdv-7. We immunized BALB/c mice with rAdMHE3 virions and produced 22 different MAbs against them, four of which showed neutralizing activity against HAdv-7 *in* *vitro*. Using an indirect enzyme-linked immunosorbent assay (ELISA) analysis and an antibody-binding-competition ELISA with Ad3EGFP, HAdv-7, and a series of chimeric adenoviral particles containing epitope mutants, we demonstrated that the four MAbs recognize the neutralization site within HVR5 of the HAdv-7 virion. Using an immunoblotting analysis and ELISA with HAdv-7, recombinant peptides, and a synthetic peptide, we also showed that the neutralizing epitope within HVR5 of the HAdv-7 virion is a conformational epitope. These findings suggest that it is feasible to use a strategy in which antigen is incorporated into the adenoviral capsid protein to generate neutralizing MAbs. This strategy may also be useful for developing therapeutic neutralizing MAbs and designing recombinant vector vaccines against HAdv-7, and in structural analysis of adenoviruses.

## Introduction

The generation of monoclonal antibodies (MAbs), especially neutralizing therapeutic MAbs, using epitope-based immunization is an attractive option. The peptide epitope represents the minimal immunogenic region of a protein antigen and epitope-based immunization allows the exquisite direction and control of the immune response [1]. However, epitope-based immunization generally results in a poor rate of MAb generation because simple peptides are poorly immunogenic and T cells must be potently stimulated and immunological memory elicited. Although several alternative approaches have been developed, including adjuvant-based strategies, conjugation with lipopeptide, direct delivery to dendritic cells, and particulate delivery systems, novel and effective methods for epitope delivery are still required [1], [2], [3].

The incorporation of an antigen into the adenovirus capsid protein is a strategy used previously to develop antibody responses against several vaccine targets [4], [5], [6], [7], [8], [9], [10], [11]. This strategy offers several potential advantages. First, when the capsid-incorporated antigen is processed via the exogenous pathway, a strong humoral response is produced, similar to the response generated by native adenoviral capsid proteins [9]. Second, incorporating immunogenic peptides into the adenovirus capsid may circumvent preexisting anti-adenoviral immunity and permit the vector to be administered repeatedly, thus allowing the response to be boosted [12], [13], [14], [15]. Third, some antigens incorporated into the adenovirus capsid may present conformations similar to those of the parental antigens [12]. With these advantages, we consider that this strategy should be developed to improve the current schemes used to generate neutralizing monoclonal antibodies (MAbs), especially for certain poorly immunogenic antigens or those that are difficult to prepare.

In our previous study, we have developed an epitope-delivery system that involves the incorporation of antigenic epitopes within the hexon hypervariable regions (HVRs) of a recombinant human adenovirus type 3 (HAdv-3)-based vector (designated “Ad3EGFP”) [16], [17], [18]. Using antisera from mice immunized with a series of chimeric viruses, we found that HVR5 of HAdv-7 may contain the major type-specific neutralizing epitope [18]. Moreover, a chimeric virus designated “rAdMHE3”, which was generated by replacing HVR5 of Ad3EGFP with HVR5 of HAdv-7, was successfully obtained [18]. Therefore, in this study, we used the strategy in which antigen is incorporated into the adenoviral capsid protein to develop HAdv-7-neutralizing MAbs with rAdMHE3 virions. We investigated whether neutralizing MAbs were able to be generated against the foreign epitope that was incorporated into the HVRs of the adenoviral capsid. Our study provides useful information for the development of therapeutic neutralizing MAbs and the design of recombinant vector vaccines against HAdv-7, and for the structural analysis of adenoviruses.

## Methods and Materials

### Ethics statement

All the experiments involving animal procedures in this study were reviewed and approved by the First Affiliated Hospital of Guangzhou Medical University Ethics Committee. The animal experiments were conducted in strict accordance with the recommendations of the Guide for the Care and Use of Laboratory Animals of the National Institutes of Health. To minimize animal suffering after viral challenge, the mice were monitored each day and mice that failed to gain weight or met the end-point criteria were humanely euthanized with pentobarbital sodium.

### Virus strains, cells, and animals

**Figure 1:**
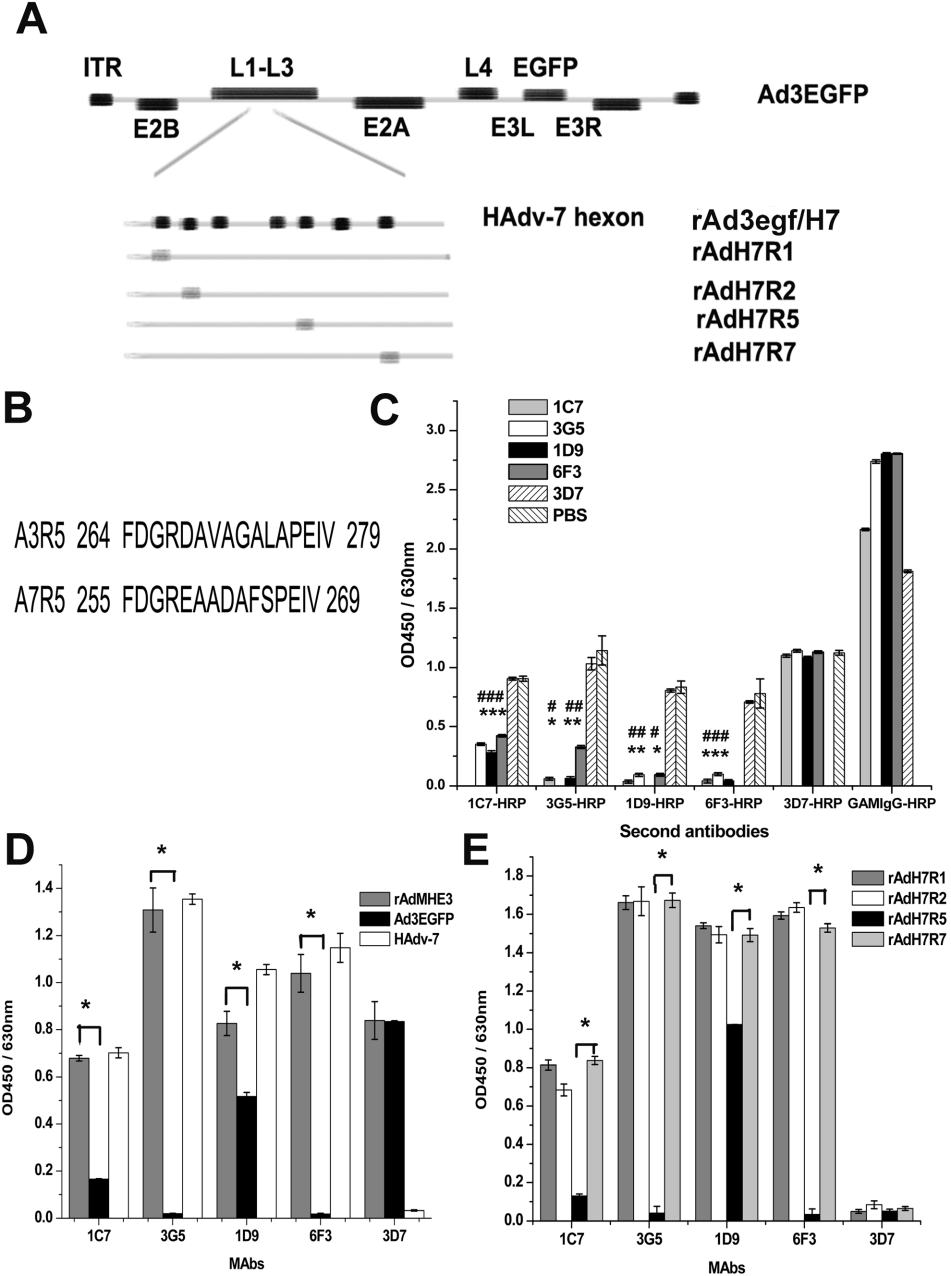
Identification of HAdv-7-neutralizing MAbs. (A) Schematic depiction of four rAd3/H7-based chimeric-hexon vectors. Epitopes derived from the HAdv-3 hexon (R1, R2, R5, and R7) are shown as gray boxes [18]. (B) HVR5 sequences of HAdv-3 and HAdv-7. (C) Analysis of an antibody-binding-competition ELISA. 96-well plates were coated with purified rAdMHE3 virions and reacted with HRP-labeled MAbs after saturation with the first MAb. 3D7, PBS, and HRP-conjugated goat anti-mouse IgG (H+L) secondary antibody (GAMIgGHRP) were used as the controls. When the binding sites were saturated with the neutralizing MAb 1C7, 3G5, 1D9, or 6F3, the subsequent binding of any other neutralizing antibody was significantly inhibited compared with the subsequent binding in the 3D7 saturation group or the PBS group. For the binding signal comparisons between each saturation group with the 3D7 saturation group, *P<0.001. For the binding signal comparisons between each saturation group with the PBS group, ^#^ *P*<0.001. (D) Indirect ELISA analysis of MAbs binding to purified wild-type or recombinant virus particles. 3D7 is one of the 22 MAbs isolated from mice vaccinated with rAdMHE3 virions. We used 3D7 here as a control. *P<0.001. (E) Indirect ELISA of MAbs binding to purified epitope mutants. *P<0.001. Each experiment was repeated independently at least three times, and the mean values and standard deviations (indicated by the error bars) are shown.

The following strains of adenovirus used in this study were obtained previously and maintained in our laboratory [16], [18], [19]: HAdv-7 GZ08 strain (GenBank accession no. GQ478341), recombinant adenovirus Ad3EGFP encoding a HAdv-3 GZ01 genome (GenBank accession no. DQ099432) and enhanced green fluorescent protein (EGFP) within an E3 region deletion (Fig. 1A), chimeric adenovirus rAdMHE3 generated by replacing HVR5 of Ad3EGFP with HVR5 of HAdv-7 (Fig. 1B), hexon chimeric adenovirus rAd3egf/H7 generated by replacing the hexon gene of Ad3EGFP with the hexon gene of HAdv-7 GZ08 (Fig. 1A), and epitope mutants (rAdH7R1, rAdH7R2, rAdH7R5, rAdH7R7) generated by replacing the HVR1, HVR2, HVR5, or HVR7 gene of rAd3egf/H7 with the corresponding HVR gene of HAdv-3 GZ01 (Fig. 1A).

Sub-lines of HEp-2 and AD293 cells (human epidermoid carcinoma cell lines) purchased from the American Type Culture Collection were kept in our laboratory to culture all the adenoviruses, and were maintained in Dulbecco’s modified Eagle’s medium (DMEM; Gibco, USA) supplemented with 100 IU/ml penicillin, 100 IU/ml streptomycin, and 10% fetal bovine serum [16], [19]. Standard CsCl gradient centrifugation was used to purify the adenoviral particles, as previously described [20]. The titers of the viral particles (VPs) were determined spectrophotometrically with a conversion factor of 1.1×10^12^ VPs per absorbance unit at 260 nm (19). BALB/c mice (6–8 weeks) were purchased from the Animal Center at Sun Yat-sen University, and housed under pathogen-free conditions.

### Generation of MAbs directed against rAdMHE3

BALB/c mice were primed intraperitoneally with 5×10^9^ purified rAdMHE3 virions per mouse in Freund’s complete adjuvant. After 2 weeks, their blood was collected from their tail veins and the serum titers against the rAdMHE3 virions were determined with an indirect enzyme-linked immunosorbent assay (ELISA). The mice were then boosted twice intraperitoneally with 10^10^ rAdMHE3 virions per mouse in Freund’s incomplete adjuvant at 2-week intervals. Three days before they were killed (at about 6 weeks after the third immunization), the mice were boosted intravenously with 2×10^10^ rAdMHE3 virions per mouse in phosphate-buffered saline (PBS). The mice were then killed and their spleen cells harvested and fused with SP2/0 myeloma cells. Mouse monoclonal antibodies against rAdMHE3 were then produced and selected as described previously [21]. In brief, the culture supernatants of the hybridomas were screened by ELISA with rAdMHE3 virions. Positive hybridomas were cloned by limiting dilution, and ascites were generated by injecting the hybridoma cells into mice primed with Freund’s incomplete adjuvant. The ascites titers against rAdMHE3 were then determined with an ELISA and 1 ml of each ascites was purified by octanoic acid–ammonium sulfate precipitation to a final volume of 150 µl. The IgG concentration was determined spectrophotometrically with a conversion factor of 1.4 mg/ml per absorbance unit at 260 nm [22]. Each purified MAb was then labeled with horseradish peroxidase (HRP) by sodium iodide oxidation at low pH.

### Virus neutralization test

For the *in vitro* adenovirus neutralization experiments, MAb ascites or antisera pretreated at 56°C for 30 min were serially diluted by 2-fold in DMEM, and 50 µl aliquots of each dilution were mixed with an equal volume of 100 TCID_50_ of wild-type adenovirus or recombinant adenovirus (HAdv-7 or Ad3EGFP). The antibody–virus mixtures were incubated for 1 h at 37°C and transferred to 96-well plates containing 85%–95% confluent monolayers of HEp-2 cells. After culture for 96 h, the monolayers were observed by microscopy and the neutralization titers were determined as the reciprocal of the highest dilution of mouse ascites or antiserum that protected the monolayers from forming a visually observable cytopathic effect (CPE).

### Antibody-binding-competition ELISA

Ninety-six-well plates (Nunc Maxisorp) were coated with rAdMHE3 virions (about 10^9^ VPs per well), washed once with 0.05% Tween 20 in PBS (PBST), and blocked for 2 h with blocking solution (30% calf serum, 5% sucrose). The first MAb ascites at a saturated dilution (1C7 1∶20, 3G5 1∶320, 1D9 1∶20, 6F3 1∶320, or 3D7 1∶20) or PBS (100 µl/well) were added and incubated for 30 min at 37°C. After the plates were washed five times with PBST, they were incubated for 30 min with a 1∶10,000 dilution of the second MAb labeled with HRP or with HRP-conjugated affinity-purified goat anti-mouse IgG (H+L) secondary antibody (GAMIgGHRP, Bio-Rad, China). 3D7 and PBS were used as the controls. The plates were then washed five times and the reaction visualized with tetramethylbenzidine (TMB) substrate, stopped with 2 M H_2_SO_4_, and analyzed at 450 nm with background subtraction at 630 nm on an ELISA plate reader (Thermo Scientific Multiskan MK3).

### Expression of recombinant protein fragments, peptide synthesis, and preparation of the antisera

HAdv-3 and HAdv-7 hexon peptides with a hexahistidine tag (designated “A3H” and “A7nH”, respectively) were expressed and purified as described previously [23]. A pGEX-4T-3 vector was used to produce a short peptide (HVR5 of HAdv-7, FDGREAADAFSPEIV) with an N-terminal glutathione S-transferase (GST) tag (designated “GST-A7R5”). GST-A7R5 was also purified as described previously [23]. The primers used for cloning were 5′-AATTCCGATGGTAGAGAAGCTGCTGACGCTTTTTCGCCTGAAATTGC-3′ and 5′-GGCCGCAATTTCAGGCGAAAAAGCGTCAGCAGCTTCTCTACCAT CGG-3′.

The peptide HVR5 of HAdv-7, designated “A7R5” (FDGREAADAFSPEIV), was synthesized and conjugated with bovine serum albumin (BSA) or keyhole limpet hemocyanin (KLH) by Jetway Co. Ltd (Guangzhou, China). The peptide was purified with high-performance liquid chromatography to 90% purity, and the identity of the peptide was confirmed by mass spectrometric analysis.

Groups of five female BALB/c mice aged 4–6 weeks were immunized with 50 µg per mouse recombinant protein A3H, A7nH, GST-A7R5, or KLH-conjugated HAdv-7 HVR5 peptide (KLH-A7R5) in Freund’s complete adjuvant by intraperitoneal injection. PBS was used as the negative control. Two booster doses were given at 2-week intervals with the same dose of antigen in Freund’s incomplete adjuvant. Blood was collected from the anesthetized mice via the retro-orbital lobe 10 days after the final immunization and transferred to microfuge tubes. The tubes were centrifuged and the sera were removed and frozen at –80±5°C. The antiserum against rAd3egf/H7 was prepared previously [18].

### Indirect ELISA analysis

For the indirect ELISA, 96-well plates (Nunc Maxisorp) were coated overnight at 4°C with VPs (about 10^9^ VPs per well), recombinant peptide (0.5 µg per well), or synthetic peptide (0.5 µg per well) in carbonate buffer (0.05 M, pH 9.6). The plates were then washed and blocked as described above. MAb ascites or antiserum (100 µl/well) diluted 1∶1000 was then added to each well and incubated for 30 min at 37°C. The plates were washed five times with PBST and incubated for 30 min with a 1∶10,000 dilution of GAMIgG-HRP. After the plates were washed five times with PBST, they were developed as described above.

### Immunoblotting analysis

HEp-2 cells were infected with the recombinant virus rAdMHE3. At 96 h postinfection, cells were harvested and freeze-thawed three times; then the virus suspensions were mixed with 5×loading buffer (10% sodium dodecyl sulfate [SDS], 5% 2-mercaptoethanol, 0.5% bromophenol blue, and 50% glycerol in 250 mM Tris-HCl [pH 6.8]) and stored at room temperature or heated for 5 min at 95°C. Samples were then separated on 8% SDS-polyacrylamide gels and electrophoretically transferred onto nitrocellulose membranes. The membranes were blocked with 5% skim-milk in Tris-buffered saline and then incubated with MAb ascites or the antiserum from mice immunized with rAdMHE3 at final dilutions of 1∶100, before they were washed again and exposed to a 1∶10,000 dilution of GAMIgG-HRP. The blot was developed with the Immobilon Western Chemiluminescence HRP Substrate (Millipore, USA).

### Statistical analysis

The data are presented as means ± standard errors. Statistical significance was determined using the Prism 5.0 software. Comparisons between two groups were made with Student’s *t* test. Comparisons among multiple groups were made with analysis of variance (ANOVA) and Bonferroni’s test. *P* values of less than 0.05 were considered statistically significant.

## Results

### Generation and identification of HAdv-7-neutralizing MAbs

Purified rAdMHE3, which was generated by replacing HVR5 of Ad3EGFP with HVR5 of HAdv-7, was used to immunize mice and screen for positive MAbs. Two weeks after each immunization, the blood of the immunized mice was collected and the serum titers against the rAdMHE3 virions were determined with ELISA. The serum titers reached 1∶10,000 after two immunizations and 1∶100,000 after the third immunization, which indicates that the humoral responses were boosted since the rAdMHE3 virions were administered repeatedly. Finally, twenty-two positive MAbs against rAdMHE3 virions were isolated. The HAdv-7-neutralizing MAbs were then screened in *in vitro* adenovirus neutralization experiments and four (1C7, 3G5, 1D9, and 6F3) with high neutralizing titers against HAdv-7, but not against Ad3EGFP, were isolated (Table 1). One MAb (3D7) with a high neutralizing titer against Ad3EGFP, but not against HAdv-7, was also isolated (Table 1). We then calculated the neutralizing titers of the five MAbs as µg/ml using the following equation: neutralizing titer (µg/ml) = IgG concentration (µg/ml)×0.150/neutralizing titer shown in Table 1. The neutralization titers of 1C7, 3G5, 1D9, 6F3, and 3D7 were calculated to be 0.98 µg/ml, 1.24 µg/ml, 2.59 µg/ml, 1.41 µg/ml, and 1.04 µg/ml, respectively.

**Table 1:** Generation of HAdv-7-neutralizing monoclonal antibodies.

MAb	Isotype	Ascites titer^a^	IgG concentration (mg/ml)^b^	Neutralization titer against viruses^c^	
				HAdv-7	Ad3EGFP
1C7	IgG1	50,000	3.36	512	<16
3G5	IgG2a	1,000,000	16.94	2048	<16
1D9	IgG1	50,000	17.71	1024	<16
6F3	IgG1	1,000,000	19.22	2048	<16
3D7^d^	IgG2a	100,000	7.13	<16	1024

a The ascites titer against rAdMHE3 is expressed as the reciprocal of the ascites dilution and was determined by indirect ELISA.

b Each ascites (1 ml) was purified by octanoic acid–ammonium sulfate precipitation to a final volume of 150 µl and the IgG concentration was determined spectrophotometrically using a conversion factor of 1.4 mg/ml per absorbance unit at 260 nm [22].

c Neutralization titer is expressed as the reciprocal of the ascites dilution and was determined as the highest dilution of ascites that protected HEp-2 cell monolayers from a visually observable CPE.

d 3D7 was one of the 22 MAbs isolated from mice vaccinated with rAdMHE3 virions. We used 3D7 here as a control.

We used an antibody-binding-competition ELISA to determine whether the four MAbs competed for the rAdMHE3 virions (Fig. 1C). The binding signals for 1C7-HRP, 3G5-HRP, 1D9-HRP, 6F3-HRP, and 3D7-HRP in each saturation group were compared with the corresponding signals in the 3D7 saturation group or PBS group, using ANOVA and Bonferroni’s test. There was no significant difference in the binding signals for 1C7-HRP, 3G5-HRP, 1D9-HRP, and 6F3-HRP between the 3D7 saturation group and the PBS group (*P*>0.05; Fig. 1C), indicating that 3D7 was a good control for this assay. When the binding sites were saturated with MAb 1C7, 3G5, 1D9, or 6F3, the subsequent binding of any other neutralizing antibody was significantly inhibited compared with the subsequent binding in the 3D7 saturation group or the PBS group (*P*<0.001; and Fig. 1C). These results suggest that the four HAdv-7-neutralizing MAbs competed for the rAdMHE3 virions.

Since the four MAbs showed neutralizing activity against HAdv-7 and HVR5 of HAdv-7 contains the neutralizing epitope [18], the four HAdv-7-neutralizing MAbs are likely to compete for the neutralization site, of which one or more individual epitopes are composed, within HVR5 of HAdv-7. To determine this, we tested the binding of the MAbs to rAdMHE3, Ad3EGFP, and HAdv-7 in indirect ELISAs. The Ad3EGFP-neutralizing antibody 3D7 was used as the control. As shown in Fig. 1D, both 3G5 and 6F3 bound to HAdv-7 and rAdMHE3 but not to Ad3EGFP. Both 1C7 and 1D9 bound to Ad3EGFP, HAdv-7, and rAdMHE3, but bound to Ad3EGFP significantly less strongly than they bound to rAdMHE3 (*P*<0.001 and *P*<0.001, respectively; Fig. 1D). We also tested the binding of the MAbs to the recombinant adenoviruses rAdH7R1, rAdH7R2, rAdH7R5, and rAdH7R7. These recombinant adenovirus are HVR mutants, of which the corresponding HVR was replaced with HAdv-3 HVR1, HVR2, HVR5, or HVR7, from rAd3egf/H7 (Fig. 1A). As shown in Fig. 1E, both 3G5 and 6F3 reacted with rAdH7R1, rAdH7R2, and rAdH7R7, but not with rAdH7R5 (Fig. 1E). Both 1C7 and 1D9 not only reacted with rAdH7R1, rAdH7R2, and rAdH7R7, but also with rAdH7R5 (Fig. 1E), although their reactivity with rAdH7R5 was significantly weaker than their reactivity with rAdH7R7 (*P*<0.001 and *P*<0.001, respectively; Fig. 1E). These results suggest that 3G5 and 6F3 recognize epitopes that are restricted to HAdv-7 HVR5, while 1C7 and 1D9 recognize epitopes which are composed of residues within EAADAFS as well as conserved regions (FDGR and PEIV) between HAdv-3 HVR5 and HAdv-7 HVR5 or additional residues outside of HAdv-7 HVR5 (Fig. 1B). Therefore, these results confirmed that the four HAdv-7-neutralizing MAbs recognize the neutralization site within HVR5 of HAdv-7.

### Characterization of the neutralizing epitope within the HVR5 region of HAdv-7

**Figure 2:**
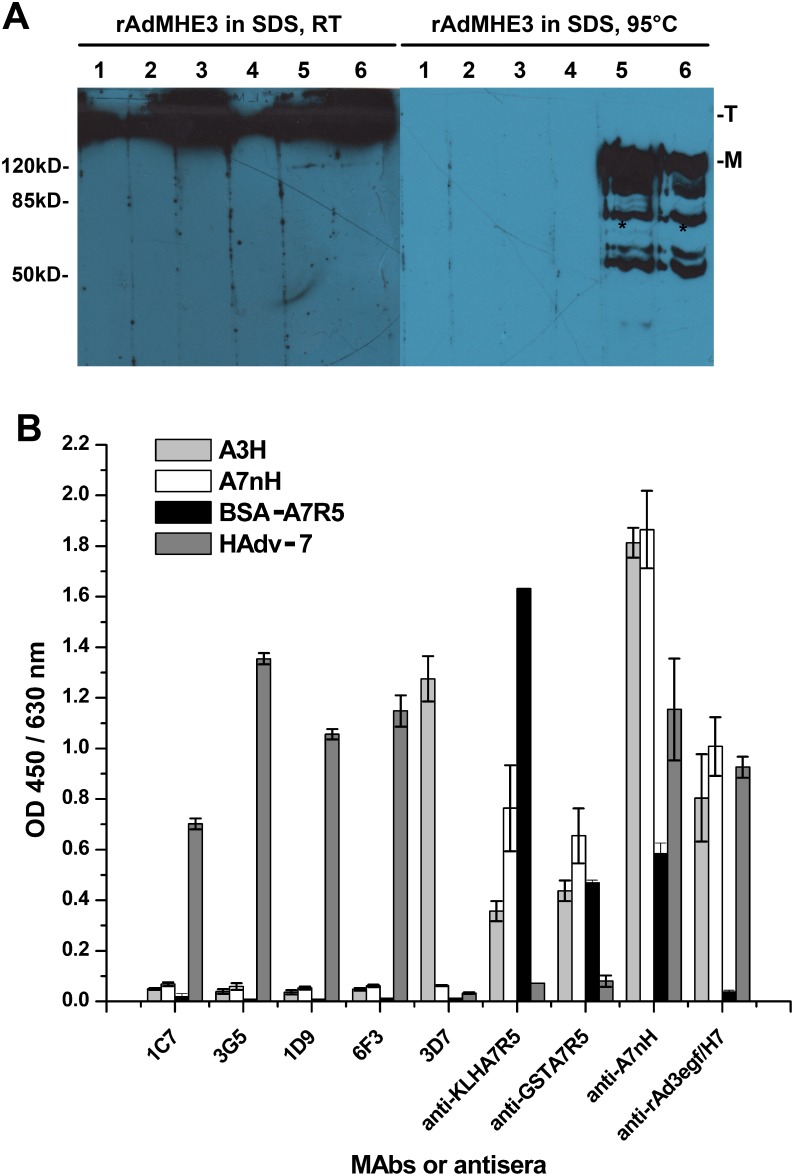
Characterization of the neutralizing epitopes within HVR5 of HAdv-7. (A) Immunoblotting analysis indicated that the four HAdv-7-neutralizing MAbs recognized hexon in trimeric form (T) at room temperature (RT), but not the hexon monomers (M) at room temperature or heated at 95°C for 5 min, in the presence of SDS. HEp-2-cell-cultured rAdMHE3 virions were harvested and stored at room temperature or heated at 95°C for 5 min in the presence of SDS. The samples were then separated by SDS-PAGE and tranferred onto nitrocellulose membranes. The membranes were then cut into individual strips and incubated with individual MAb or antiserum. After the blot being developed, the strips were spliced and their signals were exposed to two pieces of X films. The MAb 3D7, which bound to the recombinant HAdv-3 hexon protein, and the anti-rAdMHE3 serum were used as the positive controls. Lane 1, 1C7; lane 2, 3G5; lane 3, 1D9; lane 4, 6F3; lane 5, 3D7; lane 6, anti-rAdMHE3. *The rAdMHE3 virus suspensions were stored at −20°C for several months, so there may be degradation for some hexon, which may explain the minor bands of less than 120 kDa. (B) HAdv-7 virions, recombinant truncated hexon fragments of HAdv-3 (A3H) or HAdv-7 (A7nH), and BSA-conjugated synthetic HAdv-7 HVR5 peptide (BSA-A7R5) were reacted with the MAbs or different antisera in indirect ELISAs. The MAbs did not react with BSA-A7R5, A3H, or A7nH. Both anti-KLHA7R5 and anti-GSTA7R5 bound to HAdv-7 virions weakly. In addition, anti-rAd3egf/H7 bound to BSA-A7R5 weakly as well. Each experiment was repeated independently at least three times, and the mean values and standard deviations (indicated by the error bars) are shown.

To determine if the neutralizing antibodies recognized conformation-dependent or linear epitopes, we immunoblotted rAdMHE3 virions that had been electrophoresed after exposure to 2% SDS at room temperature (RT) or at 95°C. At room temperature, some hexon proteins in SDS could maintain their trimeric form [24]. The four HAdv-7-neutralizing MAbs recognized hexon in trimeric form, but not hexon monomers, in the presence of SDS (Fig. 2A), suggesting that all the four antibodies recognize conformation-dependent epitopes. This was confirmed by an ELISA analysis, in which the four MAbs did not react with the BSA-conjugated synthetic peptide A7R5 and the recombinant protein A7nH (Fig. 2B).

To determine whether the neutralizing epitope within HAdv-7 HVR5 was a conformation-dependent epitope, we collected antisera from mice immunized with rAd3egf/H7 virions, recombinant protein A7nH and GST-A7R5, and KLH-conjugated synthesized peptide KLH-A7R5, designated “anti-rAd3egf/H7”, “anti-A7nH”, “anti-GSTA7R5”, and “anti-KLHA7R5”, respectively. We then tested their respective neutralizing reactivities against HAdv-7 GZ08. We found that anti-A7nH, anti-GSTA7R5 and anti-KLHA7R5 did not neutralize HAdv-7 GZ08, whereas anti-rAd3egf/H7 did (Table 2). Further ELISA analysis was performed to test the binding of the antisera to proteins containing the HAdv-7 HVR5 motifs. As shown in Fig. 2B, both anti-KLHA7R5 and anti-GSTA7R5 bound to HAdv-7 virions weakly. In addition, anti-rAd3egf/H7 bound to BSA-A7R5 weakly as well (Fig. 2B). Since the synthesized peptide conjugated to BSA or KLH and the peptide fused in recombinant proteins may not present good conformation, these results confirmed that the neutralizing epitope within HAdv-7 HVR5 is a conformation-dependent epitope.

**Table 2:** Neutralization activity of different antisera.

Antiserum	Neutralization titer against viruses^a^	
	HAdv-7	Ad3EGFP
Anti-A7nH	<16	<16
Anti-GSTA7R5	<16	<16
Anti-KLHA7R5	<16	<16
Anti-rAd3egf/H7	>2048	128
Anti-Ad3EGFP	<16	>2048

a Neutralization titer is expressed as the reciprocal of the antiserum dilution and was determined as the highest dilution of antiserum that protected HEp-2 cell monolayers from a visually observable CPE.

## Discussion

Therapeutic MAbs are promising as biological drugs that provide protection from infectious diseases [25], [26]. Some pathogens are produced in *in vitro* culture with poor yields or cannot be propagated in cell lines. Therefore, it is difficult to generate neutralizing MAbs against these pathogens by immunization with intact pathogen particles. Epitope-based immunization is one way to generate neutralizing MAbs against these pathogens. However, simple peptides are poorly immunogenic and T cells must be potently stimulated and immunological memory elicited, so an effective epitope-delivery system is required. In the present study, we showed that HAdv-7 HVR5 displayed on carrier protein KLH or recombinant proteins GST-A7R5 and A7nH could not induce neutralizing antibodies from immunized mice (Table 2), mainly due to their inability to present good conformation of the neutralizing epitope within HVR5. Therefore, a new epitope-delivery system for conformational epitopes like the neutralizing epitope within HVR5 is required.

The strategy in which an antigen is incorporated into the adenoviral capsid protein has been described as a way to improve vaccine schemes, especially against infections requiring a strong humoral antigenic response [12]. Using this strategy, our group has developed an epitope-delivery system that involves the incorporation of antigenic epitopes within the HVRs of a recombinant HAdv-3-based vector [16], . In the present study, we used this novel epitope-delivery system to develop HAdv-7-neutralizing MAbs by incorporating HVR5 of HAdv-7 into rAdMHE3 virions. We primed the mice with 5×10^9^ or 10^10^ purified rAdMHE3 virions per mouse and found that titers of the sera from mice primed with 5×10^9^ rAdMHE3 virions were higher than those from mice primed with 10^10^ rAdMHE3 virions (data not shown). Possible explanation for this is that halving the dose for the first immunization may reduce the level of immunological tolerance. For monoclonal antibodies production using this strategy, booster immunizations are still required to allow the antibody response to be boosted. Although significant response may be detected after the second immunization, the third immunization is still required to stimulate an mature immune response. After significant serum titers are detected, an interval of 3–6 weeks is required so that new lymphoblasts in the spleen can be effectively activated after the fourth immunization before the fusion. Antigens administered intravenously are mainly presented to the spleen and the secondary lymph nodes, and administering intravenously is thought to be the ideal way for granular antigens [27]. To generate MAbs with high positive rate, therefore, in the present study we halved the dose for the first immunization and administered the rAdMHE3 virions intravenously with doubled dose for the fourth immunization. Our results showed that the humoral responses were boosted after the third immunization and finally we isolated 22 MAbs against rAdMHE3.

With the negative control virus Ad3EGFP, the NAbs against the foreign neutralizing epitopes, such as HVR5 of HAdv-7 in this paper, could be easily detected. Among the 22 MAbs against rAdMHE3 generated in the immunized mice, four HAdv-7-neutralizing MAbs were isolated that recognize the neutralization site within HVR5 of HAdv-7. These four MAbs contain both IgG1 and IgG2a subtypes (Table 1), suggesting that both Th1 and Th2 immunity had been stimulated. This finding is consistent with the previous work of Worgall et al. [5]. These data show that a strong humoral response was elicited with the capsid-incorporated antigen approach. This robust humoral response against adenoviral vectors is thought to occur because adenoviruses efficiently infect antigen-presenting cells [28], [29]. Furthermore, incorporation of antigen into the hexon protein would yield a strong anti-epitope immune response because each adenovirus virion contains 240 copies of hexon. These may explain the high positive rate of neutralizing MAb generation when this strategy is used. In conclusion, our results provide preliminary confirmation that our novel epitope-delivery system, which is based on the incorporation of antigens into the adenoviral capsid protein, is an effective delivery system for epitope-based immunization and could be feasibly developed to generate neutralizing MAbs.

Because HVR5 of HAdv-7 is an adenoviral peptide, the capsid incorporation method must still be tested on other foreign peptide antigens, to confirm that it can be used to generate neutralizing MAbs against such antigens. Currently, our group has successfully incorporated neutralizing epitopes of human enterovirus type 71, human respiratory syncytial virus, human rabies virus, and human hepatitis B virus into the virion surface of an HAdv-3-based vector. In the next step, we will generate neutralizing MAbs against these pathogens using this novel epitope-delivery system. We believe that the successful development of this strategy will improve the therapeutic neutralizing MAb generation schemes for infectious diseases.

In recent years, there have been frequent outbreaks of respiratory disease caused by the subspecies B1 adenovirus HAdv-7 in China [30], [31], [32], [33], [34]. A licensed HAdv-7 vaccine must be developed for the prevention of this disease and a neutralizing antibody for treatment of the disease. An alignment of the hexon protein sequences available in GenBank showed that the HVR5 sequence of HAdv-7 is relatively stable across time and geographic space. Therefore, the successful generation of four HAdv-7-neutralizing MAbs that recognize the neutralization site within HVR5 of HAdv-7 should allow the development of therapeutic MAbs against HAdv-7. In this study, we also found that the neutralizing epitope within HVR5 on the HAdv-7 virion is a conformational epitope. This finding will be useful for designing recombinant vector vaccines against HAdv-7 and for the structural analysis of the adenoviruses.

## References

[1] Sette A, Fikes J (2003) Epitope-based vaccines: an update on epitope identification, vaccine design and delivery. Curr Opin Immunol 15: 461–470. 12900280 10.1016/s0952-7915(03)00083-9

[2] Dudek NL, Perlmutter P, Aguilar MI, Croft NP, Purcell AW (2010) Epitope discovery and their use in peptide based vaccines. Curr Pharm Des 16: 3149–3157. 20687873 10.2174/138161210793292447

[3] Purcell AW, McCluskey J, Rossjohn J (2007) More than one reason to rethink the use of peptides in vaccine design. Nat Rev Drug Discov 6: 404–414. 17473845 10.1038/nrd2224

[4] Crompton J, Toogood CI, Wallis N, Hay RT (1994) Expression of a foreign epitope on the surface of the adenovirus hexon. J Gen Virol 75: 133–139. 7509367 10.1099/0022-1317-75-1-133

[5] Worgall S, Krause A, Rivara M, Hee KK, Vintayen EV, et al (2005) Protection against P. aeruginosa with an adenovirus vector containing an OprF epitope in the capsid. J Clin Invest 115: 1281–1289. 15841217 10.1172/JCI23135 PMC1070634

[6] Okada N, Saito T, Masunaga Y, Tsukada Y, Nakagawa S, et al (2001) Efficient antigen gene transduction using Arg-Gly-Asp fiber-mutant adenovirus vectors can potentiate antitumor vaccine efficacy and maturation of murine dendritic cells. Cancer Res 61: 7913–7919. 11691812

[7] Worgall S, Busch A, Rivara M, Bonnyay D, Leopold PL, et al (2004) Modification to the capsid of the adenovirus vector that enhance dendritic cell infection and transgene-specific cellular immune response. J Virol 78: 2572–2580. 14963160 10.1128/JVI.78.5.2572-2580.2004 PMC369215

[8] Krause A, Joh JH, Hackett NR, Roelvink PW, Bruder JT, et al (2006) Epitopes expressed in different adenovirus capsid proteins induce different levels of epitope-specific immunity. J Virol 80: 5523–5530. 16699033 10.1128/JVI.02667-05 PMC1472137

[9] Shiratsuchi T, Rai U, Krause A, Worgall S, Tsuji M (2010) Replacing adenoviral vector HVR1 with a malaria B cell epitope improves immunogenicity and circumvents preexisting immunity to adenovirus in mice. J Clin Invest 120: 3688–3701. 20811151 10.1172/JCI39812 PMC2947213

[10] Dittmer U, Brooks DM, Hasenkrug KJ (1998) Characterization of a live-attenuated retroviral vaccine demonstrates protection via immune mechanisms. J Virol 72: 6554–6558. 9658099 10.1128/jvi.72.8.6554-6558.1998 PMC109828

[11] Matthews QL, Fatima A, Tang Y, Perry BA, Tsuruta Y, et al (2010) HIV antigen incorporation within adenovirus hexon hypervariable 2 for a novel HIV vaccine approach. PLoS One 5: e11815. 20676400 10.1371/journal.pone.0011815 PMC2910733

[12] Matthews QL (2011) Capsid-incorporation of antigens into adenovirus capsid proteins for a vaccine approach. Mol Pharm 8: 3–11. 21047139 10.1021/mp100214b PMC3034826

[13] Yang Y, Li Q, Ertl HC, Wilson JM (1995) Cellular and humoral immune responses to viral antigens create barriers to lung-directed gene therapy with recombinant adenoviruses. J Virol 69: 2004–2015. 7884845 10.1128/jvi.69.4.2004-2015.1995 PMC188865

[14] Jooss K, Chirmule N (2003) Immunity to adenovirus and adeno-associated viral vectors: implications for gene therapy. Gene Ther 10: 955–963. 12756416 10.1038/sj.gt.3302037

[15] Sette A, Fikes J (2003) Epitope-based vaccines: an update on epitope identification, vaccine design and delivery. Curr Opin Immunol 15: 461–470. 12900280 10.1016/s0952-7915(03)00083-9

[16] Zhang Q, Su X, Seto D, Zheng BJ, Tian X, et al (2009) Construction and characterization of a replication-competent human adenovirus type 3-based vector as a live-vaccine candidate and a viral delivery vector. Vaccine 27: 1145–1153. 19146906 10.1016/j.vaccine.2008.12.039

[17] Tian X, Su X, Li X, Li H, Li T, et al (2012) Protection against Enterovirus 71 with Neutralizing Epitope Incorporation within Adenovirus Type 3 Hexon. PLoS One 7: e41381. 22848478 10.1371/journal.pone.0041381 PMC3407240

[18] Qiu H, Li X, Tian X, Zhou Z, Xing K, et al (2012) Serotype-specific neutralizing antibody epitopes of human adenovirus type 3 (HAdV-3) and HAdV-7 reside in multiple hexon hypervariable regions. J Virol 86: 7964–7975. 22623776 10.1128/JVI.07076-11 PMC3421688

[19] Tian X, Su X, Li H, Zhou Z, Liu W, et al (2011) Construction and characterization of human adenovirus serotype 3 packaged by serotype 7 hexon. Virus Res 160: 214–220. 21740937 10.1016/j.virusres.2011.06.017

[20] Wu H, Dmitriev I, Kashentseva E, Seki T, Wang M, et al (2002) Construction and characterization of adenovirus serotype 5 packaged by serotype 3 hexon. J Virol 76: 12775–12782. 12438602 10.1128/JVI.76.24.12775-12782.2002 PMC136697

[21] Choi EH, Kim HS, Park KH, Lee HJ (2006) Genetic heterogeneity of the hexon gene of adenovirus type 3 over a 9-year period in Korea. J Med Virol 78: 379–383. 16419117 10.1002/jmv.20550

[22] Xu ZK (1992) Shi yong dan ke long ji shu. Xi’an: Shanxi Science and Technology Press. 124 p.

[23] Tian X, Li C, Xue C, Li X, Zhou Z, et al (2013) Epitope mapping and characterization of a neutralizing monoclonal antibody against human adenovirus type 3. Virus Res 177: 189–193. 24018287 10.1016/j.virusres.2013.08.013

[24] Fortsas E, Petric M, Brown M (1994) Electrophoretic migration of adenovirus hexon under non-denaturing conditions. Virus Res 31: 57–65. 7513116 10.1016/0168-1702(94)90071-x

[25] Desoubeaux G, Daquet A, Watier H (2013) Therapeutic antibodies and infectious disease, Tours, France, November 20–22, 2012. MAbs 5: 626–632. 23883703 10.4161/mabs.25300 PMC3851213

[26] Berry JD, Gaudet RG (2011) Antibodies in infectious diseases: polyclonals, monoclonals and niche biotechnology. N Biotechnol 28: 489–501. 21473942 10.1016/j.nbt.2011.03.018 PMC7185793

[27] Herbert WJ (1978) Mineral oil adjuvants and the immunization of laboratory animals. In: Weir DM;editors. Handbook of Experimental Immunology, 3^rd^ ed. Oxford: Blackwell Scientific Publications.

[28] Labow D, Lee S, Ginsberg RJ, Crystal RG, Korst RJ (2000) Adenovirus vector-mediated gene transfer to regional lymph nodes. Hum Gene Ther 11: 759–769. 10757355 10.1089/10430340050015653

[29] Zhang Y, Chirmule N, Gao GP, Qian R, Croyle M, et al (2001) Acute cytokine response to systemic adenoviral vectors in mice is mediated by dendritic cells and macrophages. Mol Ther 3: 697–707. 11356075 10.1006/mthe.2001.0329

[30] Yu P, Ma C, Nawaz M, Han L, Zhang J, et al (2013) Outbreak of acute respiratory disease caused by human adenovirus type 7 in a military training camp in Shaanxi, China. Microbiol Immunol 57: 553–560. 23734976 10.1111/1348-0421.12074 PMC7168384

[31] Tang L, Wang L, Tan X, Xu W (2011) Adenovirus serotype 7 associated with a severe lower respiratory tract disease outbreak in infants in Shaanxi Province, China. Virol J 8: 23. 21241515 10.1186/1743-422X-8-23 PMC3030507

[32] Tsou TP, Tan BF, Chang HY, Chen WC, Huang YP, et al (2012) Community Outbreak of Adenovirus, Taiwan, 2011. Emerg Infect Dis 18: 1825–1832. 23092602 10.3201/eid1811.120629 PMC3559173

[33] Lai CY, Lee CJ, Lu CY, Lee PI, Shao PL, et al (2013) Adenovirus serotype 3 and 7 infection with acute respiratory failure in children in Taiwan, 2010–2011. PLoS One 8: e53614. 23326469 10.1371/journal.pone.0053614 PMC3542335

[34] Guo L, Gonzalez R, Zhou H, Wu C, Vernet G, et al (2012) Detection of three human adenovirus species in adults with acute respiratory infection in China. Eur J Clin Microbiol Infect Dis 31: 1051–1058. 21964587 10.1007/s10096-011-1406-8 PMC7087767

